# Severe septicemia, necrotizing fasciitis, and peritonitis due to *Vibrio vulnificus* in a patient undergoing continuous ambulatory peritoneal dialysis: a case report

**DOI:** 10.1186/s12879-015-1163-x

**Published:** 2015-10-14

**Authors:** Chang Seong Kim, Eun Hui Bae, Seong Kwon Ma, Soo Wan Kim

**Affiliations:** Department of Internal Medicine, Chonnam National University Medical School, 42 Jebongro, Gwangju, 501-757 South Korea

**Keywords:** *Vibrio vulnificus*, Septicemia, Peritonitis, Necrotizing fasciitis, Peritoneal dialysis

## Abstract

**Background:**

Chronic kidney disease, including end-stage renal disease, has been identified as a possible risk factor for primary septicemia and wound infection by *Vibrio vulnificus*. However, cases of severe septicemia, necrotizing fasciitis, and peritonitis caused by *V. vulnificus* in patients undergoing continuous ambulatory peritoneal dialysis (CAPD) have not been described. We report a case of severe septicemia, necrotizing fasciitis, and peritonitis due to *V. vulnificus* in a patient undergoing CAPD after ingesting raw seafood.

**Case presentation:**

A 37-year-old woman undergoing CAPD was admitted to the emergency room due to general weakness, fever, diarrhea, and abdominal pain. Although empirical intraperitoneal antibiotics were administered for the diagnosis of CAPD-related peritonitis, her fever did not subside. On hospital day 3, she had hemorrhagic bullae on both lower legs. We evaluated her recent food history, and found that she ate raw seafood before admission. She underwent emergency fasciotomy on the suspicion of necrotizing fasciitis by *V. vulnificus* infection. Finally, *V. vulnificus* was confirmed by 16S ribosomal ribonucleic acid gene sequencing using blood and peritoneal effluent fluid cultures*.* The administration of intraperitoneal ceftazidime and intravenous ciprofloxacin/ceftriaxone was continued for 4 weeks, and the patient completely recovered.

**Conclusions:**

Suspicion of *V. vulnificus* infection in vulnerable patients who ingest raw seafood is essential for prompt diagnosis, which could significantly improve patient outcomes.

## Background

*Vibrio vulnificus* is a species of gram-negative, motile, curved, rod-shaped pathogenic bacteria recovered from estuary, marine, and brackish environments [1]. It was first identified as a new *Vibrio* species pathogenic to humans in 1976 [2]. *V. vulnificus* infection is one of the few foodborne illnesses with an increasing incidence [3]. The main clinical manifestations of *V. vulnificus* infections are gastrointestinal illness, primary septicemia, and wound infections. Even with prompt diagnosis and aggressive therapy, the severity of infection is high, with >90 % of infected patients requiring hospitalization and a case-fatality rate of 30–50 % [1, 3–5].

Almost all patients with sepsis due to *V. vulnificus* are immunocompromised, and the common underlying conditions are alcoholic liver disease, hepatitis B or hepatitis C or viral-related cirrhosis, diabetes mellitus, and steroid use [6]. Pre-existing renal disease has been especially regarded as a risk factor for *V. vulnificus* infection. Renal disease might account for up to 7–12 % of primary septicemia cases [1, 5]. There have been 2 previous case reports of *V. vulnificus* peritonitis after consuming or handling raw seafood in patients undergoing continuous ambulatory peritoneal dialysis (CAPD) [7, 8]. However, cases of severe septicemia, necrotizing fasciitis, and peritonitis caused by *V. vulnificus* in patients undergoing CAPD have not been described. Here, we report a case of severe septicemia, necrotizing fasciitis, and peritonitis due to *V. vulnificus,* which was identified by 16S ribosomal ribonucleic acid (rRNA) gene sequencing, in a patient undergoing CAPD after ingesting raw seafood. Informed consent was obtained from the patient and the study protocol was approved by the Institutional Review Board of Chonnam National University Hospital, South Korea (CNUH-EXP-2015-129). This study was conducted according to the principles of the Declaration of Helsinki.

## Case presentation

A 37-year-old woman with end-stage renal disease (ESRD) secondary to systemic lupus nephritis was admitted to the emergency room due to general weakness, fever, diarrhea, and abdominal pain. She had been undergoing CAPD for 15 years. On physical examination, she was found to have abdominal and rebound tenderness, but no skin wound. On admission, her body temperature was 38.8 °C, pulse was 101 beats/min, and blood pressure was 80/40 mmHg. Redness or discharge at the exit site of the peritoneal catheter was not observed. The patient had the following laboratory findings (reference ranges are indicated within parentheses): white blood cell (WBC) count of 5.3 × 10^9^/L (4.8–10.8 × 10^9^/L), hemoglobin level of 8.6 g/dL (12–18 g/dL), impaired liver function as shown by aspartate and alanine transaminase levels of 48 U/L and 16 U/L, respectively (10–37 U/L), C-reactive protein level of 17.5 mg/dL (0–0.3 mg/dL), procalcitonin level of 4.23 ng/mL (0–0.5 ng/mL), iron level of 50 μg/dL (29–164 μg/dL), transferrin saturation of 43.4 % (25–50 %), and ferritin level of 3207 μg/L (4.6–274 μg/L). The peritoneal effluent fluid was cloudy with a WBC count of 560 × 10^6^/L and 55 % polymorphonuclear leukocytes. After performing peritoneal fluid and blood cultures, the patient was treated empirically with intraperitoneal cefazolin (600 mg) and ceftazidime (1 g) once daily. Two days after initiating antibiotic therapy, the abdominal pain subsided and peritoneal effluent fluid was cleared. However, her body temperature remained at 39.1 °C. On hospital day 3, she had severe painful leg edema abruptly. Moreover, she had cellulitis with ecchymosis and progressive hemorrhagic bullae on both lower legs. Shortly after, her blood pressure decreased to 60/40 mmHg. We could conduct any radiological investigations due to cardiovascular instability. She was then admitted to the intensive care unit. We briefly evaluated her recent food history, and found that she consumed salted baby octopuses and undercooked cockles few days before admission. On the suspicion of necrotizing fasciitis by *V. vulnificus* infection, she underwent emergency bedside fasciotomy. Wound cultures were performed during the fasciotomy, but showed no bacteria growth. On hospital day 4, peritoneal effluent fluid and blood cultures performed at admission revealed *V. vulnificus.* Therefore, we decided to perform a more accurate identification using 16S rRNA gene sequencing.

Genomic deoxyribonucleic acid was extracted from the cultivated microorganism using proteinase K. The 16S rRNA gene was successfully amplified with universal primers (forward: 5′-AGTTTGATCCTGGCTCAG-3′; reverse: 5′-GTATTGCCGCGGCTGCTG-3′) using Biometra T3000 (Analytik Jena, Göttingen, Germany) and sequenced using Applied Biosystems 3130 lx (Applied Biosystems, Foster City, CA, USA). The 16S rRNA gene sequences were 100 % homologous to *V. vulnificus* (GenBank accession number: KP223908.1), which was confirmed with nucleotide basic local alignment search tool analysis. *V. vulnificus* showed sensitivity to cefotaxime, ciprofloxacin, and ceftazidime, intermediate sensitivity to cefazolin, and resistance to cefoxitin using the broth microdilution methods. Therefore, intraperitoneal cefazolin was discontinued, but intravenous ciprofloxacin (200 mg twice a day) and cefotaxime (1 g every 24 h) were administered in addition to intraperitoneal ceftazidime (1 g once daily) after dose adjustment for renal insufficiency. After 7 days of treatment, the peritoneal effluent WBC count decreased to 3 × 10^6^/L. Furthermore, the absence of bacterial growth was confirmed with follow-up blood and peritoneal fluid cultures. The intraperitoneal and intravenous antibiotics were continued for 4 weeks. The patient underwent additional debridement for granulation tissues in both lower legs after emergency fasciotomy, and her wound completely healed after 9 months. The patient had been advised to avoid consumption of raw and uncooked seafood, and use separate cutting boards and knives for seafood and non-seafood. Furthermore, she was instructed to wear gloves when handling raw oysters or shellfish.

## Conclusions

*V. vulnificus* is an opportunistic human pathogen transmitted from seawater, raw oyster, and shellfish. The spectrum of disease can vary from gastroenteritis to severe septicemia and necrotizing fasciitis.

Patients with certain underlying medical conditions are susceptible to *V. vulnificus* infections. Patients with alcoholic liver disease, diabetes mellitus, gastrointestinal and hematologic disorder, malignancy, and iron overload states have a higher risk of infection [9]. Moreover, kidney diseases, including ESRD, are regarded as other potential contributing risk factors for *V. vulnificus* infection. Functional abnormalities of neutrophils, monocytes, dendritic cells, and lymphocytes have been observed in uremia, which makes patients prone to specific life-threatening infections [10]. In addition, dialysis patients have frequently elevated iron levels because of blood transfusion or iron supplement for significant anemia. Iron is believed to facilitate *V. vulnificus* infection by enhancing the growth of the organism and possibly reducing the activity of neutrophils [11]. *V. vulnificus* grows rapidly when transferrin saturation exceeds 70 % [12]. In this context, a relatively high transferrin saturation in our patient might be an additional risk factor for infection.

*V. vulnificus* infection has not been well described in patients undergoing peritoneal dialysis. Interestingly, there are only 2 case reports of *V. vulnificus* peritonitis after consuming or handling seafood in patients undergoing CAPD, and these patients completely recovered after oral and intraperitoneal antibiotic treatments [7, 8]. Similarly, our patient showed peritonitis after ingesting raw seafood. The proportion of *V. vulnificus* infections in Korea and the United States has rapidly increased during the last decade due to the increased consumption of raw fish or shellfish [1, 3]. Although previous reports of *V. vulnificus* peritonitis, including our report, were conducted in Asia, patients undergoing CAPD and live in an area with high incidence of *V. vulnificus* infection should avoid raw or undercooked seafood to prevent infection. A remarkable finding about our case was that peritonitis was followed by severe septicemia and necrotizing fasciitis. We could presume that *V. vulnificus* in the peritoneal fluid might penetrate the peritoneum or bowel wall into the blood stream, which results in septicemia. Therefore, peritonitis might be an important clinical manifestation of *V. vulnificus* infection in peritoneal dialysis patients. Furthermore, according to our findings, patients undergoing peritoneal dialysis are highly susceptible to various degrees of *V. vulnificus* infection from peritonitis to septicemia and necrotizing fasciitis.

An array of phenotypic and genomic techniques has become available for the identification of *Vibrio* species. However, *Vibrio* and other closely related species show similar phenotypic features and are not easily distinguished biochemically [13]. The identification of *Vibrios* isolated from the aquaculture environment has been imprecise and requires many biochemical and/or physiological tests. Although the 16S rRNA gene sequencing method may be less reliable because many different species within the genus *Vibrio* may contain identical 16S rRNA gene sequences, it remains one of the most commonly used methods for bacterial identification [14]. 16S rRNA gene sequencing can be used in hospitals to identify *V. vulnificus* that are difficult to identify using phenotypic and biochemical methods.

Moreover, the recommended antibiotic therapy for *V. vulnificus* is tetracycline family (e.g. tetracycline, doxycycline, and minocycline) plus third-generation cephalosporin [15]. Alternative antibiotics therapies are cefotaxime or ciprofloxacin [1]. In a recent retrospective study, fluoroquinolones were found to be the best option for the antibiotic treatment of necrotizing fasciitis caused by *V. vulnificus* [16]. Therefore, fluoroquinolones or tetracycline plus third-generation cephalosporin could be prescribed empirically in a patient with severe *V. vulnificus* infection.

Despite reports of high mortality from *V. vulnificus* infection, our patient recovered without complication. Necrotizing fasciitis is a life threatening soft tissue infection with a high mortality rate, which requires emergent surgical fasciotomy, debridement, and broad-spectrum antibiotic treatment when the diagnosis is confirmed [17]. Consequently, early surgical intervention was performed and adequate antibiotics treatments were administered to our patient; these strategies might dramatically improve the patient’s survival. Finally, suspicion of *V. vulnificus* infection in vulnerable patients who ingest raw seafood is essential for prompt diagnosis, which could significantly improve patient outcomes.

### Consent

Written informed consent for publication of this case and any accompanying images was obtained from the patient. A copy of the written consent is available for review by the Editor of this journal.

## Abbreviations

CAPD: continuous ambulatory peritoneal dialysis
rRNA: ribosomal ribonucleic acid
ESRD: end-stage renal disease
WBC: white blood cell
